# Pooled analysis of case-control studies on acoustic neuroma diagnosed 1997–2003 and 2007–2009 and use of mobile and cordless phones

**DOI:** 10.3892/ijo.2013.2025

**Published:** 2013-07-22

**Authors:** LENNART HARDELL, MICHAEL CARLBERG, FREDRIK SÖDERQVIST, KJELL HANSSON MILD

**Affiliations:** 1 Department of Oncology, University Hospital, SE-701 85 Örebro;; 2 Department of Radiation Physics, Umeå University, SE-90187 Umeå, Sweden

**Keywords:** vestibular schwannoma, risk factors, cell phones, wireless phones, ionzing radiation

## Abstract

We previously conducted a case-control study of acoustic neuroma. Subjects of both genders aged 20–80 years, diagnosed during 1997–2003 in parts of Sweden, were included, and the results were published. We have since made a further study for the time period 2007–2009 including both men and women aged 18–75 years selected from throughout the country. These new results for acoustic neuroma have not been published to date. Similar methods were used for both study periods. In each, one population-based control, matched on gender and age (within five years), was identified from the Swedish Population Registry. Exposures were assessed by a self-administered questionnaire supplemented by a phone interview. Since the number of acoustic neuroma cases in the new study was low we now present pooled results from both study periods based on 316 participating cases and 3,530 controls. Unconditional logistic regression analysis was performed, adjusting for age, gender, year of diagnosis and socio-economic index (SEI). Use of mobile phones of the analogue type gave odds ratio (OR) = 2.9, 95% confidence interval (CI) = 2.0–4.3, increasing with >20 years latency (time since first exposure) to OR = 7.7, 95% CI = 2.8–21. Digital 2G mobile phone use gave OR = 1.5, 95% CI = 1.1–2.1, increasing with latency >15 years to an OR = 1.8, 95% CI = 0.8–4.2. The results for cordless phone use were OR = 1.5, 95% CI = 1.1–2.1, and, for latency of >20 years, OR = 6.5, 95% CI = 1.7–26. Digital type wireless phones (2G and 3G mobile phones and cordless phones) gave OR = 1.5, 95% CI = 1.1–2.0 increasing to OR = 8.1, 95% CI = 2.0–32 with latency >20 years. For total wireless phone use, the highest risk was calculated for the longest latency time >20 years: OR = 4.4, 95% CI = 2.2–9.0. Several of the calculations in the long latency category were based on low numbers of exposed cases. Ipsilateral use resulted in a higher risk than contralateral for both mobile and cordless phones. OR increased per 100 h cumulative use and per year of latency for mobile phones and cordless phones, though the increase was not statistically significant for cordless phones. The percentage tumour volume increased per year of latency and per 100 h of cumulative use, statistically significant for analogue phones. This study confirmed previous results demonstrating an association between mobile and cordless phone use and acoustic neuroma.

## Introduction

Acoustic neuroma or vestibular schwannoma is a benign tumour in the eighth cranial nerve that leads from the inner ear to the brain. It is a slowly growing tumour in the auditory canal and expands gradually into the cerebellopontine angle with potential compression of vital brain stem centres. It tends to be encapsulated and grows in relation to the auditory and vestibular portions of the nerve. This tumour type does not undergo malignant transformation. Tinnitus and hearing problems are the usual first symptoms of acoustic neuroma. Although it is a benign tumour it may cause persistent disabling symptoms after treatment such as loss of hearing and tinnitus that severely affect the daily life.

Acoustic neuroma is a rare tumour. The average age-standardised incidence rates ranged during 1987–2007 from 6.1 per 1,000,000 in Finnish men to 11.6 in Danish men. Women in Sweden had the lowest average rate of 6.4 per 1,000,000 and the highest rate, 11.6, was found in Denmark ( 1 ) . The incidence increased significantly during the time period 1987–2007 when all Nordic countries (Denmark, Finland, Norway and Sweden) and both genders were combined, +3.0% per year, 95% confidence interval (CI) = +2.1 to 3.9%.

The aetiology of acoustic neuroma is not well known. Risk factors such as exposure to ionising radiation during childhood ( 2 ) and loud noise ( 3 ) have been suggested. Neurofibromatosis 2 is one established risk factor for acoustic neuroma with 90–95% lifetime risk ( 4 ) .

During calls when a wireless phone (mobile phone or cordless phone; DECT) is held close to the head the eighth cranial nerve is expected to receive relatively high exposure to radiofrequency electromagnetic fields (RF-EMF). Thus, there is a particular concern about increased risk for acoustic neuroma due to exposure to RF-EMF emissions during use of these devices. Results for long-term use of wireless phones and the risk for acoustic neuroma have been published by the Hardell group ( 5 , 6 ) and by the WHO Interphone study group; only mobile phone use was published for Interphone ( 7 ) . Both sets of studies provided corroborative results, demonstrating an association between acoustic neuroma and exposure to RF-EMF from wireless phones. We have recently summarised and discussed these results ( 8 , 9 ) .

In May 2011, the International Agency for Research on Cancer (IARC) at WHO evaluated the carcinogenic effect of RF-EMF on humans. The evaluation included radiation from mobile phones and from other devices that emit similar non-ionising electromagnetic fields. The conclusions stated that there were positive associations between exposure to radiofrequency radiation from wireless phones and glioma, and acoustic neuroma. It was concluded that RF-EMF is a Group 2B, i.e. a ‘possible’ human carcinogen ( 10 , 11 ) .

In order to obtain results relating to longer-term use of wireless phones we decided to perform a new case-control study on brain tumours encompassing study subjects during the time period 2007–2009. The ethics committee also approved this new study.

The results for malignant brain tumours and meningioma are being published separately. This report presents the results for acoustic neuroma. Since the cases in this new study were few (n=73), we decided to make a pooled analysis for the two study periods 1997–2003 and 2007–2009.

## Materials and methods

### Wireless technology

Wireless technology has been used in Sweden since the early 1980s. Initially, analogue phones (NMT; Nordic Mobile Telephone System) were used, but this system was finally closed down in 2007. Since the early 1990s the market has been increasingly dominated by digital GSM phones. In 2003 the third generation of mobile phones, 3G or UMTS (Universal Mobile Telecommunication System), was introduced in Sweden. Currently the fourth generation, 4G (Terrestrial 3G), is being established. Nowadays, mobile phones are used more than landline phones in Sweden ( 12 ) . Worldwide, an estimated 5.9 billion mobile phone subscriptions were reported at the end of 2011 by the International Telecommunication Union ( 13 ) .

Desktop cordless phones (DECT) have been used in Sweden since 1988, first using analogue 800–900 MHz RF fields, but since early 1990s using a digital 1900 MHz system. They are very common, overtaking telephones connected to landlines. These devices also emit RF-EMF radiation when used and should be given equal consideration with mobile phones when human health risks are evaluated.

### Inclusion criteria

This report is based on results from two study periods, 1997–2003 and 2007–2009. The same methods were used for both periods including similar questions on use of mobile and cordless phones. All studies were of the case-control design and included both men and women who were alive. Cases were reported to us from the cancer registries. The diagnosis was based on histopathology in all cases. Tumour localisation (side of head) was based on reports to the cancer registries and medical records, which were obtained after informed consent from the patients.

Cases with both benign and malignant brain tumours were included in the study. For each case one control matched on age in 5-year groups and gender, living in the same geographical region as the respective case, was drawn from the population registry. They were assigned the same year as the diagnosis of the respective case as cut-off in assessment of exposure. All these controls were used in the analysis of the results for acoustic neuroma.

The results for the time period 1997–2003, which included the age group 20–80 years, have been published previously and further details can be found in these reports [Hardell *et al* ( 5 , 8 , 14 ) ]. Cases and controls aged 20–80 years at the time of diagnosis living in certain geographical areas in Sweden, as presented in those publications, were included during that time period.

Our new study included cases aged 18–75 years at the time of diagnosis during 2007–2009. Again, the diagnosis was verified by histopathology in all cases. They were reported to us from cancer registries and the whole of Sweden was now included. For administrative reasons the Gothenburg region could only be included for the years 2008 and 2009.

For both study periods the responsible physician was contacted for permission before the case was included. Medical records including computer tomography (CT) and/or magnetic resonance imaging (MRI) were used for calculation of tumour volume.

### Exposure assessment

The questionnaire was similar for both study periods. Use of wireless phones, i.e. both mobile and cordless phones, was assessed by a self-administered questionnaire supplemented by a phone interview. The questionnaire also contained a number of other questions on e.g. occupation, exposure to different agents, smoking habits, medical history including hereditary risk factors, and exposure to ionizing radiation. These questions were also supplemented over the phone by the interviewer. A structured protocol was used for all questions during the interviews.

The ear that had been most regularly used during calls with mobile and/or cordless phone was assessed by separate questions; >50% of the time for one side, or equally for both sides. The matched control was assigned the same side as the tumour of the respective case in the series of studies. The whole procedure was conducted without knowledge of exposure status. Use of the wireless phone was defined as ipsilateral (≥50% of the time) or contralateral (<50% of the time) in relation to tumour side.

Each questionnaire received a unique Id-number that did not disclose whether it was a case or a control. Thus, case or control status was not disclosed to the interviewer or during further data processing. All information was coded and entered into a database. Case or control status was not disclosed until the statistical analyses.

### Statistical methods

All analyses were done using StataSE 12.1 (Stata/SE 12.1 for Windows; StataCorp., College Station, TX). Odds ratios (OR) and 95% confidence intervals (CI) were calculated using unconditional logistic regression analysis including the whole control sample (i.e. matched to both malignant and benign cases) to increase the power of the study.

Latency period (time between first exposure and diagnosis) was defined using year of first use of a wireless phone and year of diagnosis (the same year for the matched control). The cumulative number of hours of use was calculated using number of years and average time used per day. Use in a car with external antenna was disregarded; so was use of a handsfree device. We adopted a minimum latency period of one year (≤1 year) for exposure, less than that was included in the unexposed category. The same year as for each case’s diagnosis was used for the corresponding control as the cut-off for exposure accumulation. Note that latency was calculated separately for the respective phone type or combination of phones that were analysed.

Adjustment was made for the matching variables gender, age (as a continuous variable), and year of diagnosis. In addition, adjustment was made for socio-economic index (SEI) divided into four categories (blue-collar worker, white-collar worker, self-employed, no work), since an association between white-collar work and brain tumours has been reported ( 15 ) . Latency was analysed using five time periods, >1–5 years, >5–10 years, >10–15 years, >15–20 years and >20 years. Cumulative use of the various phone types and combinations was analysed in quartiles based on the distribution of total use of wireless phones among the controls. Latency and cumulative use were also analysed as continuous variables (per year of latency, per 100 h cumulative use) to further explore the dose-response relations. Laterality was not analysed for the whole group of wireless phone users since the side could differ for mobile phone and cordless phone for the same person.

Restricted cubic splines were used to visualize the relationship between cumulative use and latency of wireless phones and acoustic neuroma. Adjustment was made for the same variables as in the logistic regression. Four knots were used at the 5th, 35th, 65 and 95th percentiles as suggested by Harrell ( 16 ) . P-value for non-linearity was estimated by testing if the coefficient of the second and third spline was equal to zero, using the Wald test. Tumour volume was estimated using the ellipsoid formula:

$$\frac{4}{3}\pi (\frac{D_{1}}{2}\times \frac{D_{2}}{2}\times \frac{D_{3}}{2})$$

(D _1_ , D _2_ , D _3_ , diameters in the three axis). Change of tumour volume per year of latency and per 100 h of cumulative use was analysed using linear regression analysis, adjusted for age and gender. The volumes were log-transformed to normalize the distribution. The percentage changes were calculated from the β coefficients in the model, using the expression: (e ^β-coefficient-1^ ) × 100.

## Results

Of the 338 cases with acoustic neuroma, 316 (93%) answered the questionnaire; 141 were men and 175 women. Of the 4,038 controls, 3,530 (87%) participated, 1,492 men and 2,038 women. The mean age was 52 years for cases (median 53, range 23–80) and 54 years for all controls (median 55, range 19–80).

Table I summarises the results for acoustic neuroma and use of wireless phones. Analogue phones yielded OR = 2.9, 95% CI = 2.0–4.3 increasing to OR = 7.7, 95% CI = 2.8–21 in the longest latency group >20 years.

Use of digital 2G phones yielded a total OR = 1.5, 95% CI = 1.1–2.1 with somewhat higher OR in the longest latency group >15 years. The results for digital 3G were based on low numbers with short latency period. Overall, mobile phone use gave a statistically significant increased risk with the highest risk in the longest latency group >20 years yielding OR = 4.5, 95% CI = 2.1–9.5.

Cordless phone use gave OR = 1.5, 95% CI = 1.1–2.1, with higher risk in the longest latency group >20 years with OR = 6.5, 95% CI = 1.7–26, but based on low numbers. Wireless phone use overall gave OR = 1.5, 95% CI = 1.1–2.0 increasing with latency >20 years to OR = 4.4, 95% CI = 2.2–9.0.

Table II summarises the results for use of wireless phones in relation to tumour side. For all studied phone types except digital 3G, somewhat higher ORs were calculated for ipsilateral wireless phone use than for contralateral.

Cumulative use of wireless phones was analysed in quartiles ( Table III ). Note that for the various phone types the cumulative time was counted for use of the specific phone, but for the category ‘mobile phones’ all types of mobile phones were included, and for ‘wireless phones’ use of cordless phones was also included. In general, the highest ORs were found in the fourth quartile with >1,486 h cumulative use. Mobile phone use in the fourth quartile gave OR = 2.6, 95% CI = 1.5–4.4 (p trend = 0.052), cordless phone use yielded OR = 1.9, 95% CI = 1.1–3.2 (p trend = 0.11) and wireless phone use overall gave OR = 2.2, 95% CI = 1.5–3.4 (p trend = 0.03).

The highest increase in risk per 100 h cumulative use and per year of latency was found for analogue phones, OR = 1.049, 95% CI = 1.022–1.076 and OR = 1.098, 95% CI = 1.062–1.136, respectively ( Table IV ). There was a statistically non-significant increase for cordless phone use. The digital types of wireless phones gave statistically significantly increased risk per 100 h cumulative use, OR = 1.006, 95% CI = 1.0001–1.013, and per year of latency, OR = 1.035, 95% CI = 1.0003–1.071. Overall, use of wireless phones gave statistically signficant increased risks per 100 h of cumulative use and per year of latency.

Gender-specific analyses yielded similar results. Cumulative use of wireless phones gave OR = 2.9, 95% CI = 1.5–5.6 for men in the fourth quartile and OR = 1.9, 95% CI = 1.1–3.4 for women; thus the results for both genders were statistically significant with 95% CI overlapping ORs (data not shown).

**Figure 1.**
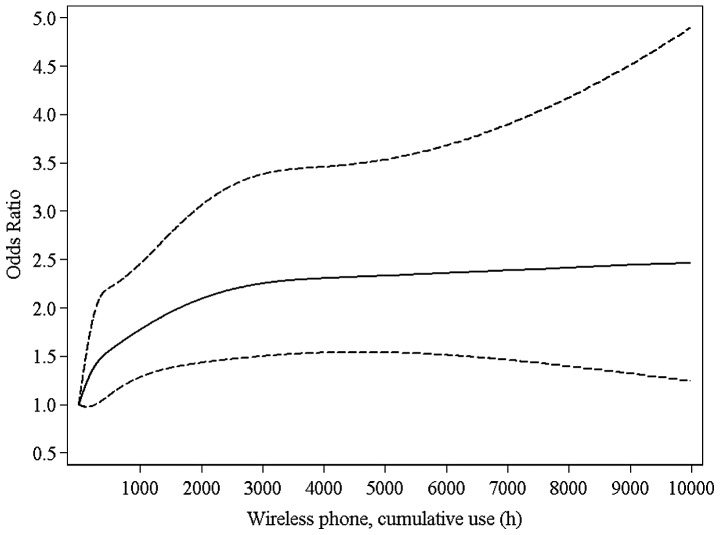
Restricted cubic spline plot of the relationship between cumulative use of wireless phones and acoustic neuroma. The solid line indicates the OR estimate and the broken lines represent the 95% CI. Adjustment was made for age at diagnosis, gender, SEI-code and year of diagnosis.

**Figure 2.**
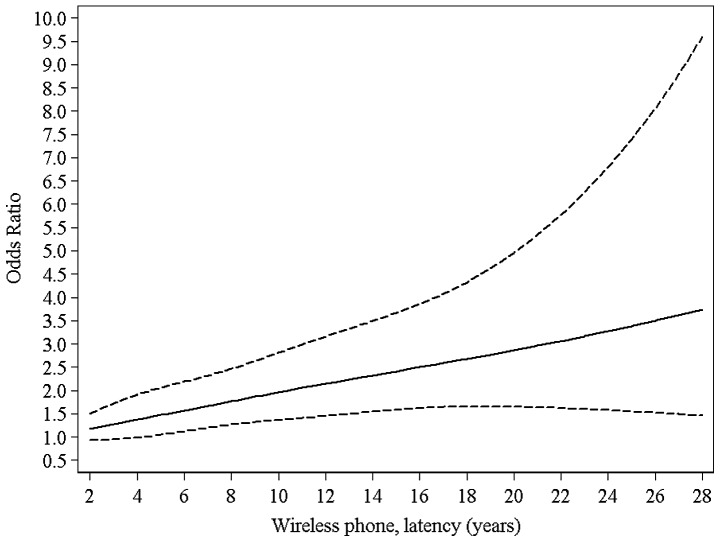
Restricted cubic spline plot of the relationship between latency of wireless phones and acoustic neuroma. The solid line indicates the OR estimate and the broken lines represent the 95% CI. Adjustment was made for age at diagnosis, gender, SEI-code and year of diagnosis.

Fig. 1 illustrates the results for cumulative use of wireless phones using the restricted cubic splines method. The sharpest increase in risk was seen up to approximately 3,000 h of cumulative use; up to 10,000 h the increase was less (p, nonlinearity = 0.01). Fig. 2 demonstrates a linear relationship (p, non-linearity = 0.60) between increasing risk and latency using data up to 28 years from first use of a wireless phone before tumour diagnosis.

For 218 cases with acoustic neuroma, tumour volume could be calculated on the basis of information in available CT/MRI reports. There was no statistically significant difference according to gender or age, although for cases aged >53 years (cut-off at median age) a somewhat larger volume was calculated than for lower age (median 4.2 versus 2.0 cm ^3^ ). Percentage tumour volume change per year of latency and per 100 h of cumulative use increased for all types of wireless phones and was statistically significant for analogue phones ( Table V ). The results for digital 3G phone was based on only seven cases so calculations were not meaningful.

## Discussion

### Main findings

The main result of this study was an association between use of wireless phones and acoustic neuroma. Increased risk was found for all studied phone types with the highest ORs in the longest latency period. Formally, the highest OR overall was calculated for digital mobile phones of the third generation (3G) , but this was not statistically significant and was based on low numbers of exposed cases. Since this technology is rather new, data on long-term use are lacking.

It should be noted that most subjects had used several phone types. Increased risks were found for use of only analogue and only digital (2G) mobile phones (data not shown). Most of these calculations were hampered by numbers too low to permit meaningful interpretation of the results. Nevertheless, in the >10 year latency group, only analogue mobile phone use gave OR = 4.2, 95% CI = 0.8–21 and only digital 2G mobile phone use gave OR = 3.6, 95% CI = 1.2–11. The corresponding result for only cordless phone use was OR = 1.5, 95% CI = 0.3–7.3. A high risk was calculated for use of both mobile and cordless phones in the latency group >20 years yielding OR = 6.2, 95% CI = 2.8–14.

Most of the RF-EMF emissions from a handheld phone are absorbed on the side of the brain on which the phone is used (ipsilateral), with the highest dose in the area where acoustic neuroma develops ( 17 ) . We found higher ORs for ipsilateral wireless phone use, but increased risks were also calculated for contralateral use. One contributing factor to the latter finding could be that hearing deficit is an early clinical sign of acoustic neuroma; the subjects might change the ear for phone use due to that circumstance.

In our present study, cumulative use of wireless phones was divided into quartiles depending on cumulative use of wireless phones overall among controls. For wireless phones the highest overall risk was found in the fourth quartile >1,486 h of cumulative use. This corresponds to approximately 25 min wireless phone use per day for 10 years. There was a statistically significant trend (p=0.03) for increasing cumulative use of wireless phones overall, but the trend was of borderline statistical significance for mobile phones (p=0.052). The OR showed a statistically significant increase per 100 h of cumulative use and per year of latency for both mobile and wireless phone use. Cordless phone use also increased the OR per 100 h of cumulative use and per year of latency.

Tumour volume increased per year of latency and per 100 h of cumulative use of wireless phones. The result was statistically significant for analogue phones, in accordance with overall findings of higher risk for use of that phone type. It should be noted that the increase in tumour volume was higher for ipsilateral use of mobile phones of the digital 2G type and for cordless phones than for contralateral use of the respective type. This ought to make the findings biologically more relevant (data not shown).

### Strengths and limitations

In our new case-control study for the period 2007–2009 there were few cases with acoustic neuroma (n=73; eight did not participate). Statistical analysis of the results was less meaningful although the whole control sample (n=1,368) for the study period could be used. We decided to include our previous study period 1997–2003 and make a pooled analysis. Thus, 243 additional cases and 2,162 additional controls were included in the pooled analysis. This was justified by the fact that a similar questionnaire was used for both study periods. Assessment of use of both mobile and cordless phones was the same including the similar protocol for supplementary phone interviews regarding unclear facts or to verify exposures. Furthermore, in the statistical analysis, adjustment was made for year of diagnosis, gender, age and SEI-code.

Recall and observational bias might be an issue in case-control studies. We investigated in more detail the possibility of that in one of our previous studies ( 18 ) . Reporting a previous cancer or if a relative helped to fill in the questionnaire did not change the results. Potential observational bias during phone interviews was analysed by comparing change of exposure in cases and controls after these interviews. No statistically significant differences were found, showing that our results are unlikely to be explained by observational bias. To further validate exposure in the present study we used meningioma cases (n=1,624) as the referents to the acoustic neuroma cases (n=315). Similar results were found. Thus, wireless phone use gave in total (>1 year latency) OR = 1.4, 95% CI = 1.005–1.9, and in the latency group >20 years OR = 3.2, 95% CI = 1.5–6.8 with meningioma cases as referents. The corresponding results with population based controls were OR = 1.5, 95% CI = 1.1–2.0 and OR = 4.4, 95% CI = 2.2–9.0, respectively ( Table I ). These results clearly show that the results in this study can not be explained by recall or observational bias.

In our previous study on acoustic neuroma ( 5 ) a diagnostic head X-ray was associated with an overall increased risk; OR = 3.1, 95% CI = 2.2–4.2 (unpublished data). The risk increased to OR = 7.5, 95% CI = 3.4–16 for >3 occasions of X-ray investigations with >1 year latency. However, there was no interaction with mobile phone use (p=0.73), cordless phone use (p= 0.95), or wireless phone use (p= 0.81). In the present study X-ray investigations of the head were again assessed. These data are to be analysed further, but in view of our previous results an interaction with wireless phone use is unlikely.

Certainly some X-ray investigations might be tumour-related, but using >10 year latency, X-ray of the head gave OR = 4.9, 95% CI = 1.5–16, indicating it is a risk factor for acoustic neuroma. Dental X-ray investigations did not increase the risk for acoustic neuroma in the 1997–2003 time period study: OR = 0.6, 95% CI = 0.3–1.4 (n=236 cases, 2,124 controls; missing data for seven cases and 38 controls); there was no dose-response relationship. The literature on dental and head X-ray investigations and the risk for acoustic neuroma is scanty. In the German part of Interphone, medical ionising radiation gave OR = 0.97, 95% CI=0.54–1.75 for acoustic neuroma ( 19 ) . In a study from Brazil on 44 acoustic neuroma patients and 104 controls, exposure to >1 cranial X-ray investigation gave OR = 4.55; 95% CI = 1.10–19.2 ( 20 ) .

Frequent dental X-ray investigations were associated with an increased risk for acoustic neuroma encompassing 343 patients who underwent Gamma Knife surgery and 343 matched control patients with degenerative spinal disorders ( 21 ) . Head and neck CT was associated with a statistically significantly decreased risk, which casts doubt on the study methods including selection of controls.

Loud noise has been suggested as a risk factor for acoustic neuroma ( 3 ) . In the questionnaire we asked for exposure to ‘extremely high noise’, and the results are available for the study period 1997–2003. This gave OR = 1.4, 95% CI = 0.97–1.9, increasing somewhat to OR = 1.5, 95% CI = 1.01–2.2 in the >10 years latency group. However, there was no interaction with use of wireless phones (p=0.71) or the different phone types.

One strength of our whole study was that we included only cases with a histopathological diagnosis of a brain tumour. This was because we wanted a valid diagnosis of the brain tumour for separate analysis depending on tumour type. If necessary, the histopathological reports were supplemented by records from pathology departments around the country after informed consent from the subject. Thus, we were able to classify all brain tumours on the basis of WHO codes. Neurofibromatosis type II was identified in two cases with acoustic neuroma. Exclusion of these cases did not change the results.

Stereotactic radiosurgery is one option for treatment of acoustic neuroma, especially smaller ones ( 22 , 23 ) . Obviously in these cases the diagnosis is made by CT and MRI without histopathology. However, exclusion of cases with only clinical diagnosis is unlikely to have biased the results, since criteria for treatment are not expected to be related to habits of wireless phone use.

One advantage of this study was the high response rate among both cases and controls. The response rate was 93% (n=316) among the finally included cases with acoustic neuroma. Of the controls, 87% (n=3,530) answered the questionnaire. In the Interphone study on acoustic neuroma ( 7 ) lower response rates were obtained for both cases and controls; see below. To ensure that results are as valid as possible, a high response rate is always necessary. In fact, non-responding controls in Interphone tended to be less frequent users of mobile phones than participating controls, leading to underestimation of the risk ( 24 – 26 ) .

### Results from other studies

A case-case study on acoustic neuroma and mobile phone use was conducted in Japan ( 27 ) . The cases were identified during 2000–2006 at 22 participating neurosurgery departments. The diagnosis was based on histopathology or CT/MRI imaging. Of 1,589 cases 816 (51%) agreed to participate and answered a mailed questionnaire. A total of 787 cases were included in the final analysis. Two datasets were analysed, one comprising 362 cases with no tumour-related symptoms one year before diagnosis, and the other comprising 593 cases with no symptoms five years before diagnosis. Cases with ipsilateral mobile phone use were regarded as exposed and those with contralateral use were assumed to be unexposed and were treated as the reference category. Overall, no increased risk was found. However, for average daily call duration >20 min with reference date one year, risk ratio (RR) = 2.74, 95% CI = 1.18–7.85 increased to RR = 3.08, 95% CI = 1.47–7.41 with reference date five years before diagnosis. Unfortunately, no results were given for cumulative hours of use over the years. For cordless phones no increased risk was found but the analysis was not very informative.

In the Interphone study, 1,121 (82%) acoustic neuroma cases participated, range 70–100% by centre ( 7 ) . Of the controls 7,658 (53%) completed the interviews, range 35–74% by centre. The final matched analysis (1:1 or 1:2) comprised 1,105 cases and 2,145 controls. Overall no increased risk was found censoring exposure at one year or at five years before the reference date, OR = 0.85, 95% CI = 0.69–1.04 and OR = 0.95, 95% CI = 0.77–1.17, respectively. Cumulative number of hours of ipsilateral mobile phone use ≥1,640 h up to one year before the reference date gave OR = 2.33, 95% CI = 1.23–4.40 and contralateral use OR = 0.72, 95% CI = 0.34–1.53 for acoustic neuroma ( 7 ) . Cumulative number of hours of ipsilateral mobile phone use ≥1,640 hours up to five years before the reference date gave OR = 3.53, 95% CI = 1.59–7.82, and for contralateral use OR = 1.69, 95% CI = 0.43–6.69. The risk increased further for cumulative ipsilateral use ≥1,640 h with start ≥10 years before the reference date to OR = 3.74, 95% CI = 1.58–8.83. Contralateral use in that group yielded OR = 0.48, 95% CI = 0.12–1.94; however, this was based on only four exposed cases and nine exposed controls. Overall, OR = 1.93, 95% CI = 1.10–3.38 was obtained for long-term use with start ≥10 years before the reference date and cumulative call time ≥1,640 h.

We conducted a meta-analysis on mobile phone use and its association with acoustic neuroma based on results by the Hardell group ( 5 ) and the Interphone study ( 7 ) . The analysis was based on published results by Interphone since we do not have access to their database. Our results were recalculated to these exposure groups. A random-effects model was used based on a test for heterogeneity in the overall (≥10 years and ≥1,640 h) groups. For the latency group ≥10 years, the highest risk was obtained for ipsilateral use: OR = 1.81, 95% CI = 0.73–4.45. The risk increased further for cumulative use ≥1,640 h yielding OR = 2.55, 95% CI = 1.50–4.40 for ipsilateral use ( 8 ) .

In the study by Han *et al* ( 21 ) regular mobile phone use was statistically significant more common among the cases (p= 0.006). The adjusted OR for ≥10 years’ mobile phone use was 1.29, 95% CI = 0.69–2.43 (crude OR = 2.20, 95% CI = 1.43–3.39). Regarding cordless phone use the adjusted OR for ≥10 years use was 1.07, 95% CI = 0.51–2.21 (crude OR = 1.40, 95% CI = 0.84–2.35). However, not all statistically significant confounders were included in the adjusted model (residency excluded) and no results were given for wireless phone use in total. The authors noted that they had insufficient information on mobile phone use. The results for cordless phones were not discussed in detail.

An increased risk for acoustic neuroma associated with reported use of mobile phone was found in a study from UK ( 28 ) . Ever use gave in the 10+ years group RR = 2.46, 95% CI = 1.07–5.64 with increasing risk with duration of use (trend p=0.03). The study was limited by e.g. mobile phone use only at baseline, no details on handedness use, no information on tumour laterality and no assessment of use of cordless phones.

In conclusion, this study confirmed previous results of an association between use of mobile and cordless phones and acoustic neuroma. The risk increased with time since first use. For use of both mobile and cordless phones the risk was highest in the longest latency group. Tumour volume increased per 100 h of cumulative use and years of latency for wireless phones. Using the meningioma cases as reference entity gave similar results as with population based controls indicating that the results could not be explained by recall or observational bias.

## Figures and Tables

**Table I.** Odds ratio (OR) and 95% confidence interval (CI) for acoustic neuroma based on 316 cases and 3,530 controls. ^a^

Latency	Analogue OR, CI (Ca/Co)	Digital (2G) OR, CI (Ca/Co)	Digital (UMTS, 3G) OR, CI (Ca/Co)	Mobile phone, total OR, CI (Ca/Co)	Cordless phone OR, CI (Ca/Co)	Digital type OR, CI (Ca/Co)	Wireless phone OR, CI (Ca/Co)
Acoustic neuroma (n=316)							
Total, >1 year	2.9	1.5	3.9	1.6	1.5	1.5	1.5
	2.0–4.3 (86/558)	1.1–2.1 (173/2,014)	0.4–35 (7/141)	1.2–2.2 (200/2,148)	1.1–2.1 (156/1,724)	1.1–2.0 (216/2,393)	1.1–2.0 (227/2,472)
>1–5 years	2.2	1.4	4.1	1.3	1.5	1.4	1.2
	1.2–4.0 (16/87)	0.996–2.0 (80/714)	0.5–36 (7/127)	0.9–1.8 (65/674)	1.05–2.1 (72/653)	1.01–1.9 (93/796)	0.8–1.6 (72/748)
>5–10 years	3.2	1.8	-	2.3	1.6	1.6	1.9
	2.0–5.2 (33/137)	1.1–2.8 (56/659)	(0/14)	1.6–3.3 (77/688)	1.1–2.5 (60/655)	1.1–2.3 (73/758)	1.3–2.7 (84/767)
>10–15 years	3.0	1.8	-	2.1	1.4	1.6	2.0
	1.6–5.7 (16/113)	0.97–3.4 (28/471)	(0/0)	1.3–3.5 (34/476)	0.8–2.6 (19/294)	0.97–2.8 (38/584)	1.3–3.2 (44/578)
>15–20 years	3.5	1.8	-	2.1	0.5	1.1	1.7
	1.5–8.5 (9/107)	0.8–4.2 (9/170)	(0/0)	1.02–4.2 (12/196)	0.1–2.1 (2/109)	0.5–2.5 (9/242)	0.9–3.3 (13/253)
>20 years	7.7	-	-	4.5	6.5	8.1	4.4
	2.8–21 (12/114)	(0/0)	(0/0)	2.1–9.5 (12/114)	1.7–26 (3/13)	2.0–32 (3/13)	2.2–9.0 (14/126)

a Numbers of exposed cases (Ca) and controls (Co) are given. Adjustment was made for age at diagnosis, gender, SEI-code and year of diagnosis.

**Table II.** Odds ratio (OR) and 95% confidence interval (CI) for acoustic neuroma, total, ipsilateral and contralateral exposure. ^a^

	All			Ipsilateral			Contralateral		
	Ca/Co	OR	95% CI	Ca/Co	OR	95% CI	Ca/Co	OR	95% CI
Analogue	86/558	2.9	2.0–4.3	54/252	2.9	1.9–4.6	29/184	2.5	1.4–4.2
Digital (2G)	173/2,014	1.5	1.1–2.1	108/865	1.7	1.1–2.4	62/684	1.3	0.9–2.1
Digital (UMTS, 3G)	7/141	3.9	0.4–35	3/70	1.9	0.2–20	3/45	3.6	0.3–38
Mobile phone, total	200/2,148	1.6	1.2–2.2	123/920	1.8	1.3–2.6	73/729	1.5	0.98–2.2
Cordless phone	156/1,724	1.5	1.1–2.1	101/766	1.8	1.2–2.6	52/565	1.2	0.7–1.8

a Numbers of exposed cases (Ca) and controls (Co) are displayed. Adjustment was made for age at diagnosis, gender, SEI-code and year of diagnosis. Ipsilateral, ≥50% use of the phone on the same side as the tumour was located. Contralateral, <50% use of the phone on the same side as the tumour was located.

**Table III.** Odds ratio (OR) and 95% confidence interval (CI) for dose-response between use of wireless phones and acoustic neuroma. ^a^

Quartile	Analogue OR, CI (Ca/Co)	Digital (2G) OR, CI (Ca/Co)	Digital (UMTS, 3G) OR, CI (Ca/Co)	Mobile phone, total OR, CI (Ca/Co)	Cordless phone OR, CI (Ca/Co)	Digital type OR, CI (Ca/Co)	Wireless phone OR, CI (Ca/Co)
First quartile	2.5	1.5	9.1	1.6	1.2	1.3	1.2
	1.6–3.9 (42/304)	1.04–2.1 (83/885)	0.9–89 (5/47)	1.1–2.2 (91/920)	0.8–1.8 (36/478)	0.9–1.9 (59/618)	0.8–1.7 (57/641)
Second quartile	3.1	1.2	1.5	1.5	1.6	1.3	1.5
	1.8–5.5 (23/146)	0.7–2.0 (30/467)	0.1–26 (1/54)	0.9–2.3 (37/492)	1.03–2.3 (49/534)	0.9–2.0 (49/583)	1.02–2.2 (56/596)
Third quartile	4.2	2.2	2.7	2.4	2.1	1.9	1.9
	2.1–8.4 (14/82)	1.3–3.6 (38/388)	0.2–47 (1/31)	1.5–3.8 (42/416)	1.3–3.2 (47/451)	1.3–2.8 (58/613)	1.3–2.8 (58/617)
Fourth quartile	6.6	2.1	-	2.6	1.9	2.1	2.2
	2.6–17 (7/26)	1.2–3.9 (22/274)	(0/9)	1.5–4.4 (30/320)	1.1–3.2 (24/261)	1.4–3.3 (50/579)	1.5–3.4 (56/618)

a Numbers of exposed cases (Ca) and controls (Co) are displayed. Adjustment was made for age at diagnosis, gender, SEI-code and year of diagnosis. First quartile, 1–122 h; second quartile, 123–511 h; third quartile, 512–1,486 h; fourth quartile, >1,486 h. p, trend: analogue, p=0.16; digital (2G), p=0.08; digital (UMTS, 3G), p=0.14; mobile phone, total, p=0.052; cordless phone, p=0.11; digital type, p=0.07; wireless phone, p=0.03.

**Table IV.** Odds ratio (OR) and 95% confidence interval (CI) for acoustic neuroma per 100 h of cumulative use and per year of latency. ^a^

Type of phone	Per 100 h cumulative use		Per year of latency	
	OR	95% CI	OR	95% CI
Analogue	1.049	1.022–1.076	1.098	1.062–1.136
Digital (2G)	1.008	0.998–1.018	1.043	0.998–1.089
Digital (UMTS, 3G)	0.915	0.724–1.157	0.992	0.670–1.468
Mobile phone, total	1.009	1.001–1.017	1.060	1.031–1.089
Cordless phone	1.007	0.998–1.016	1.028	0.992–1.065
Digital type	1.006	1.0001–1.013	1.035	1.0003–1.071
Wireless phone	1.008	1.002–1.014	1.056	1.029–1.085

a Adjustment was made for age at diagnosis, gender, SEI-code and year of diagnosis.

**Table V.** Percentage change in tumour volume per year of latency and per 100 h of cumulative use. ^a^

Type of phone	n	Change in volume per year of latency (%)	95% CI	p-value	Change in volume per 100 h of cumulative use (%)	95% CI	p-value
Analogue	61	+7.4	+1.0 to 14.2	0.02	+10.3	+2.4 to 18.7	0.01
Digital, 2G	116	+2.1	−4.1 to 8.6	0.52	+1.4	−0.6 to 3.5	0.18
Digital, UMTS, 3G	7	-	-	-	-	-	-
Mobile phone, total	137	+3.6	−1.1 to 8.6	0.13	+1.7	−0.1 to 3.5	0.06
Cordless phone	104	+4.2	−3.8 to 13.0	0.31	+1.2	−1.1 to 3.6	0.31
Wireless phone	153	+3.6	−1.1 to 8.6	0.13	+1.0	−0.1 to 2.2	0.08

a Adjustment was made for age at diagnosis and gender.
